# The Influence of the Occupational Exposure to Heavy Metals and Tobacco Smoke on the Selected Oxidative Stress Markers in Smelters

**DOI:** 10.1007/s12011-014-9984-9

**Published:** 2014-05-01

**Authors:** Milena Ściskalska, Marta Zalewska, Agnieszka Grzelak, Halina Milnerowicz

**Affiliations:** 1 Department of Biomedical and Environmental Analysis, Faculty of Pharmacy, Wroclaw Medical University, Borowska 211, 50-556 Wrocław, Poland; 2 Students Scientific Society at the Department of Biomedical Environmental Analysis, Faculty of Pharmacy, Wroclaw Medical University, Borowska 211, 50-556 Wrocław, Poland

**Keywords:** AOPP, Cadmium, Heavy metals, MDA, Tobacco smoke, 8-OHdG

## Abstract

The aim of the study was to verify if there is any association between exposure to Cu, Zn, Cd, Pb, As and the formation of malondialdehyde (MDA), 8-hydroxydeoxyguanosine (8-OHdG), advanced oxidation protein products (AOPP), and whether in this process cigarette smoking plays a role. The investigations were performed in the 352 smelters occupationally exposed to heavy metals and 73 persons of control group. Metals concentration was determined by atomic absorption spectrometry. MDA and AOPP concentrations were determined by spectrophotometric methods. The concentration of 8-OHdG was determined by ELISA method. It was demonstrated an increased Cu concentration in smoking smelters compared to non-smoking control group. It was noted no differences in Zn and Mg concentrations between the examined groups. Pb concentration was more than sixfold higher in the group of smoking smelters and about fivefold higher in the group of non-smoking smelters compared to the control groups (smokers and non-smokers). It was shown that Cd concentration in the blood was nearly fivefold higher in the smoking control group compared to the non-smoking control group and more than threefold higher in the group of smoking smelters compared to non-smoking. It was shown an increased As concentration (more than fourfold) and decreased Ca concentration in both groups of smelters compared to control groups. In groups of smelters (smokers and non-smokers), twofold higher MDA and AOPP concentrations, and AOPP/albumin index compared to control groups (smokers and non-smokers) were shown. Tobacco smoke is the major source of Cd in the blood of smelters. Occupational exposure causes accumulation of Pb in the blood. Occupational exposure to heavy metals causes raise of MDA concentration and causes greater increase in AOPP concentration than tobacco smoke.

## Introduction

Free radicals are not only formed continuously during normal physiological processes, but also as a result of external factors, including exposure to tobacco smoke in the case of smokers or to heavy metals in the work environment. In tobacco smoke, free radicals are responsible for cell damage and apoptosis [1]. They can cause DNA fragmentation, oxidative damage of cellular membrane lipids and proteins, and the reduction of enzyme activity through its oxidation and nitration [1–3]. The most important radicals, which have a strong toxic effect, are oxygen free radicals—superoxide anion (O_2_^•−^), hydroxyl radical (^•^OH^−^), hydrogen peroxide (H_2_O_2_), and nitrogen free radicals—peroxynitrites (NO_2_^−^) and nitrates (NO_3_^−^) [3]. Except to these radicals generated directly, tobacco smoke contains many substances, such as polycyclic aromatic hydrocarbons, heterocyclic substances, N-nitrosamines, aromatic amines, and aldehydes, which are probably the main causes of biomolecules damage [4, 5]. Components of tobacco smoke are also metals, e.g., arsenic, beryllium, nickel, chromium, cadmium, polonium, cobalt, and lead [4]. These substances have the ability to generate free radicals or catalyze reactions in which they are produced [3].

Oxidative stress can intensify lipids peroxidation. One of the most common products of lipid peroxidation is malonylodialdehyde (MDA), which is also generated in the process of biosynthesis of prostaglandins. MDA can be transmitted to distant tissues and, thanks to the possibility of forming covalent bonds with other molecules, modifies their structure, and consequently, changes its properties. MDA has high cytotoxicity, is mutagenic, and inhibits the activity of various enzymes, leading to inhibition of DNA replication, transcription, and breathing [6, 7]. MDA is considered as a marker of oxidative damage of cell membrane. It is formed from the decomposition of primary and secondary lipids peroxidation products [8, 9]. Another component of the cell which is characterized by its particular susceptibility to oxidative damage is DNA [7]. Free radicals can react with both purine and pyrimidine bases, as well as the deoxyribose. Oxidative stress can damage DNA comprising: breaking single or double strand, base modifications, deoxyribose modifications, and crosslinks formation. When DNA damage is not repaired before or during replication, this can lead to cell death, mutations, replication errors, and instability of the genome, each of which is associated with the process of carcinogenesis [6]. Of all the DNA nitrogen bases, guanine is the most susceptible to oxidation [10, 11]. Hydroxyl radical addition to the eighth position of the molecule leads to the formation of guanine modified product—8-hydroxydeoxyguanosine (8-OHdG) [11, 12]. Mutagenic character of 8-OHdG located in the DNA template is due to its potential for incorrect base pairing during replication. 8-OHdG is capable of transversion G:C → T:A. It has been shown that such transversions are common in human cancers and are especially prevalent in the p53 tumor suppressor gene. This indicates the importance of 8-OHdG as an endogenous mutagen and its potential role in tumorigenesis [13].

Exposure of proteins to reactive oxygen species results in modification of amino acid residues, what alters protein structure and function [5]. The changes in proteins, which are induced by exposure to tobacco smoke, such as an increase of carbonyl groups content in the protein molecule were shown [3, 14]. The protein oxidation can be caused also by the exposure on heavy metals, which are present in living or/and occupational environment. The broad spectrum of heavy metals toxicity, such as Pb, Cd, and As, resulting from their ability to selective interaction with the fragments of protein chain, may induce the production of oxygen free radicals [15]. This toxicity of heavy metals can be reduced by the presence of Ca and Mg—an important macronutrients, which limit the absorption of metals, such as Cd and Pb, in human and animals organisms [16, 17].

Albumin is a major protein of blood plasma, where it constitutes about 55 % of all proteins. This protein is also a major and predominant antioxidant in plasma [18]. The ability of the albumin to perform these functions has a relationship with its structure, which may be destroyed by oxidative stress. Free radicals can interact with each other or with amino acids/proteins. The modifications of amino acids lead to an increase of carbonyl groups and disulphides in the protein molecule, and reduce the number of α-helices, what results in conformational changes of proteins [3, 19, 20]. The influence on the albumin function, in particular on its ability to transport, have not only free radicals, but also heavy metal ions. Their binding to albumin is in competition with other physiological substances transported by albumin. Toxic metals, as compounds with high affinity to the hydrophobic sites of albumin, cause a conformational change of the protein molecule, the amino acids oxidation, and the weakness of the ability to binding other ligands. In addition, the heavy metal ions present in the blood cause a chelation of physiological ligands. However, the strength of the phenomenon is dependent on the type of metal. In the presence of heavy metal ions, the toxins, which are transported by albumin, are displaced from the connections with protein. This results in an increase of free toxins in the blood, having an impact on the body [21, 22].

The oxidation of proteins, including albumin, leads to the formation of permanent products, which vary in terms of structure, function, and physicochemical properties. These are known as an advanced oxidation protein products (AOPP) [23]. Although the exact structure of AOPP is not completely understood, it is known that they are mainly derivatives of oxidatively modified albumin, its aggregates and/or fragments, but also fibrinogen and lipoproteins, glycoproteins, and globulins (e.g., thyroglobulin and γ-globulins) [18, 23–26].

The aim of the study was to verify if there is any association between exposure to metals (Cu, Zn, Cd, Pb, and As) occurring in the work environment of smelters and the formation of MDA, 8-OHdG, and AOPP and whether in this process cigarette smoking plays a role. The influence of the exposure to heavy metals and tobacco smoke on the concentration of Ca and Mg also was analyzed.

## Material and Methods

### Materials

The investigations were performed in the whole blood, serum, plasma, and urine of 352 males working in the copper foundry and 73 healthy men, non-exposed occupationally to heavy metals, who were qualified as control group by clinician of primary medical care. The study protocol was approved by Local Bioethics Committee of Wroclaw Medical University (Nr KB: 469/2008). The study population was in similar age and similar BMI. Table 1 presents data concerning age, BMI, years of work in metallurgy, and cotinine concentration in serum of smelters and persons of control group divided into smokers (22 pack years of smoking) and non-smokers: 258 smoking smelters, 94 non-smoking smelters, 14 smokers of control group, and 59 non-smokers of control group. Data about smoking were obtained from direct personal interview and were verified by the determination of serum cotinine concentration—the metabolite of nicotine.

**Table 1:** Characteristics of the persons in control group and the group of smelters

	Smoking X ± SD	Non-smoking X ± SD
Control group	(*n* = 14)	(*n* = 59)
Age	34.38 ± 7.90	36.64 ± 11.49
BMI [kg/m^2^]	26.98 ± 5.48	25.75 ± 3.50
Years of work in smelter	–	–
Serum cotinine [ng/ml]	66.46 ± 12.18 ^a^	3.46 ± 3.47
Smelters	(*n* = 258)	(*n* = 94)
Age	42.06 ± 9.06	44.02 ± 8.86
BMI [kg/m^2^]	26.02 ± 3.66	27.41 ± 3.61
Years of work in smelter	18.98 ± 1.87	19.99 ± 8.82
Serum cotinine [ng/ml]	71.19 ± 12.65^a^	2.81 ± 1.94

^*a*^ *p* < 0.0001 compared to non-smoking group

*BMI* body mass index

Venous blood was collected in the morning, after 12-h fasting. Serum was obtained according to the standard procedure by taking venous blood for disposable trace element-free tubes (No. Cat 03.1524.001, Sarstedt, Germany) with serum clotting activator, left at 25 °C to complete thrombosis, and centrifuged (1,200 g/20 min). In order to obtain plasma, blood was collected into trace element-free tubes containing EDTA-K_2_ (No. Cat 04.1931.001, Sarstedt, Germany), immediately gently mixed, and centrifuged (2,500 g/15 min). The obtained serum, plasma, and whole blood were portioned and stored in sealed tubes (No. Cat. 0030102.002, Eppendorf, Germany). Blood samples of smelters and persons of control groups were stored at −80 °C until analysis.

The urine was collected in the morning, according to the standard procedure. Subjects were asked to wash their hands before supplying the urine, in order to reduce contamination. Urine samples were stored in special container containing liquid nitric acid at −20 °C until determination.

### Metals Concentration

Concentrations of metals were determined using Solaar M6 aparatus (Solaar House, Cambridge, UK). The Pb concentration in whole blood (Pb-B) was determined by graphite furnace atomic absorption spectrometry (GFAAS) in the graphite Massman cuvette, the absorbance measurement at wavelength λ = 283.3 nm, with Zieeman background correction. The reference material was used BCR-194, -195, -196, IRMM, EU. The Cd concentration in whole blood (Cd-B) and in urine (Cd-U) was determined by the same method at wavelength λ = 228.8 nm, with Zieeman background correction. The concentration of Zn and Cu in serum (Zn-S and Cu-S) was determined by flame atomic absorption spectrometry (FAAS) in air-acetylene flame at wavelength λ = 324.8 nm. The reference material was used Single-Element Zinc (Copper), standard 1000 μg/ml, CPI International. The determination of Ca and Mg concentrations in serum (Ca-S and Mg-S) was also performed by this method at wavelength λ = 422.7 nm for Ca and λ = 285.2 nm for Mg, with deuterium correction of the glass. As a reference material for these metals was used Seronorm™ Trace Element Serum (Sero, No. 201 405). The As concentration in urine (As-U) (with 65 % HNO_3_ for acidification) was determined by FAAS method with snap Phillips PU 9360, used for hydride generation. In this method, wavelength λ = 193.7 nm deuterium background correction and quartz atomization chamber were used.

### Creatinine Concentration in Urine

Urinary creatinine level was determined by the Jaffé reaction [27].

### Cotinine Concentration

Serum cotinine concentration was determined using a competitive enzyme immunoassay, the DRG Serum Cotinine ELISA kit (Cat. No. EIA-3242, DRG International, USA). The cotinine determination was carried out by the method described earlier [28, 29].

### Plasma MDA Concentration (MDA-P)

Lipid peroxidation was measured in plasma by measuring the formation of thiobarbituric acid reactive substances (TBARS), quantified as malondialdehyde (MDA) equivalents, according to the method described earlier [30, 31]. The amount of MDA was calculated using an extinction coefficient (1.56 × 10^5^ M^−1^ cm^−1^). The MDA intensity was measured by spectrophotometric method at λ = 535 nm. The concentrations of MDA were expressed as micromoles per liter in plasma.

### Serum 8-OHdG Concentration (8-OHdG-S)

To evaluate the concentration of 8-OHdG in the serum, a commercial test OxiSelect™ DNA Oxidative Damage ELISA Kit was used (Cat. No. STA-320, Cell Biolabs, Inc., USA). To 96-well plate, 100 μl of conjugate 8-OHdG/BSA was added and incubated overnight at 4 °C, and then washed with H_2_O. The next step, 200 μl blocking buffer was added and incubated at room temperature for 1 h. Fifty microliter of samples and 8-OHdG standards were added. After 10 min of incubation, monoclonal anti-8-OHdG was added (100 μl, 1 h incubation at room temperature), washed three times followed by addition of secondary antibody conjugated to horseradish peroxidase (100 μl, 1 h incubation at room temperature). The plate was washed three times with washing buffer and to each well 100 μl of substrate for peroxidase was added and incubated for 20 min. Followed by the addition of 100 μl of reaction stop solution. A spectrophotometric measurement of absorbance was performed at a wavelength λ = 450 nm. The content of 8-OHdG in the tested samples was calculated by comparison with a standard curve determined from standards treated similarly to the samples tested.

### Plasma AOPP Concentration (AOPP-P)

Determination of AOPP-P concentration was performed by spectrophotometric method developed by Witko-Sarsat et al. [26], based on the reaction of AOPP with potassium iodide in acidic conditions. To 1 ml of plasma, tenfold diluted with PBS, and to the blank sample (1 ml PBS), 50 μl of potassium iodide (99 %) and 100 μl of acetic acid (99.5 %) were added and mixed. The absorbance of samples was measured against the blank at wavelength λ = 340 nm. The results of determination were expressed in micromoles per liter chloramine T equivalents and also were converted per gram of albumin and expressed as AOPP/albumin index (μmol/g).

### Serum Albumin Concentration (Alb-S)

Alb-S concentration was determined by using the bromocresol purple (BCP) (Cat. No. 115 − 40 − 2, Sigma-Aldrich), which reacts with albumin in phosphate buffer saline (PBS) at pH = 6.8. The concentration of albumin standard (Cat. No. 70024 − 90 − 70, Sigma-Aldrich) was determined using the molar absorption coefficient (A_280 nm_^0.1 %^ = 0.5). To make the standard curve, the standard solutions at concentrations 0, 11.3, 22.6, 33.9, 45.2, 56.5 g/l were prepared, which were obtained by the dilution of albumin in PBS.

For each of the tubes, working reagent (with the final concentration of BCP 40 μmol/l), serum sample, or albumin standard were added and mixed. All samples were incubated for 5 min at 25 °C. Absorbances of the standards and of the samples were measured against the blank sample (working reagent) at wavelength λ = 603 nm. The increase in absorbance was proportional to the concentration of albumin in the sample.

### Statistical Analysis

Statistical analysis was carried out using the program Statistica 10.0. Data about examined groups, BMI, age, years of work in smeltery, and cotinine concentration, were analysed using ANOVA Kruskal−Wallis test. The concentration of metals (Cu-S, Zn-S, Pb-B, Cd-B, Cd-U, As-U, Ca-S, and Mg-S), MDA-P, 8-OHdG, AOPP-P, albumin, and the value of AOPP/albumin index also using the same test were analyzed. In order to verify the association between parameters (age, BMI, concentration of cotinine, metals, MDA-P, AOPP-P, albumin, and AOPP/albumin index), the multiple linear regression models were performed. Statistical significance was accepted for *p* < 0.05.

## Results

### The Concentrations of Metals

In this study, the concentrations of Cu, Zn, Ca, and Mg in serum; Pb and Cd in whole blood; and Cd and As in urine were determined (Table 2). In the smoking smelters, a higher concentration of Cu in serum (Cu-S) compared to non-smoking control group was shown. There was no statistically significant difference in serum Zn (Zn-S) and Mg (Mg-S) concentrations between smoking and non-smoking smelters, and between smoking and non-smoking control group. In the serum of smoking and non-smoking smelters, the decrease of Ca concentration (Ca-S) in comparison to the smokers and non-smokers in control group was noted.

**Table 2:** Influence of cigarette smoking on the metals concentrations in the group of smelters and control group

Metal	Smelters		Control group	
	Smoking (*n* = 258) X ± SD	Non-smoking (*n* = 94) X ± SD	Smoking (*n* = 14) X ± SD	Non-smoking (*n* = 59) X ± SD
Cu-S [μg/100 ml]	107.22 ± 13.22^b^	104.00 ± 13.63	102.29 ± 13.46	97.76 ± 10.66
Zn-S [μg/100 ml]	101.22 ± 23.37	97.60 ± 18.44	99.22 ± 10.67	96.97 ± 9.22
Pb-B [μg/l]	229.91 ± 127.75^a,b^	177.91 ± 125.17^a,b^	37.95 ± 14.15	31.01 ± 7.91
Cd-B [μg/l]	1.52 ± 0.98^b,c^	0.46 ± 0.33^a^	1.52 ± 0.77^b^	0.32 ± 0.10
Cd-U [μg/g creatinine]	0.83 ± 0.66^a,b^	0.56 ± 0.41	0.44 ± 0.26	0.32 ± 0.20
As-U [μg/g creatinine]	12.76 ± 7.69^a,b^	13.01 ± 9.56^a,b^	3.14 ± 2.16	2.73 ± 1.24
Ca-S [μg/ml]	89.81 ± 8.03^a,b^	90.77 ± 8.34^a,b^	97.59 ± 4.64	100.02 ± 4.02
Mg-S [μg/ml]	21.08 ± 2.01	20.62 ± 1.10	19.76 ± 1.64	20.70 ± 1.70

^*a*^ *p* < 0.05 compared to smoking control group

^b^ *p* < 0.05 compared to non-smoking control group

^c^ *p* < 0.05 compared to non-smoking smelters

*Cu-S* Cu concentration in serum; *Zn-S* Zn concentration in serum; *Pb-B* Pb concentration in the blood; *Cd-B* Cd concentration in the blood; *Cd-U* Cd concentration in urine; *As-U* As concentration in urine; *Ca-S* Ca concentration in serum; *Mg-S* Mg concentration in serum

In both smoking and non-smoking smelters, a significant increase in the concentration of Pb in the blood (Pb-B) compared to the control group (smokers and non-smokers) was observed. In the group of smoking smelters, 6-fold increase in Pb-B concentration in comparison to the smoking control group and 7.4-fold higher in relation to the non-smoking control group were observed. In the group of the non-smoking smelters, 5.7-fold higher Pb-B concentration compared to the non-smoking control group was observed. The concentration of Cd in the blood (Cd-B) was significantly higher at smokers (both the smelters and the control group) compared to non-smokers. In the blood of smokers (both the smelters and the control group), 3.3-fold higher Cd concentration compared to the non-smoking smelters and 4.8-fold higher compared to the non-smoking control group were observed.

In the group of smoking smelters, 2.5-fold higher concentration of Cd in urine (Cd-U) compared to the non-smoking control group was observed. In the urine of smoking and non-smoking smelters, As concentration (As-U) was significantly higher compared to control groups (both smokers and non-smokers). In the smelters (smoking and non-smoking), more than 4-fold higher of As-U concentration in comparison to the smoking control group and more than 4.6-fold higher compared to the non-smoking control group were observed.

### MDA-P, 8-OHdG-S, and AOPP-P Concentrations as Markers of Lipids Peroxidation, Oxidatively Modified DNA, and Protein Oxidation

In the groups of smelters (smokers and non-smokers), 2-fold higher MDA-P concentration compared to control groups (respectively, smokers and non-smokers) was shown (Table 3). However, there was no statistically significant difference in 8-OHdG-S concentration between smoking and non-smoking smelters, and between smoking and non-smoking control group (Table 3).

**Table 3:** Influence of cigarette smoking on MDA, 8-OHdG, AOPP, albumin concentrations, and AOPP/albumin index in the group of smelters and control group

Parametr	Smelters		Control group	
	Smoking (*n* = 258) X ± SD	Non-smoking (*n* = 94) X ± SD	Smoking (*n* = 14) X ± SD	Non-smoking (*n* = 59) X ± SD
MDA-P [μmol/l]	1.56 ± 0.76^a,b^	1.54 ± 0.65^a,b^	0.74 ± 0.12	0.80 ± 0.24
8-OHdG-S [ng/ml]	2.73 ± 0.31	2.63 ± 0.41	2.60 ± 0.48	2.61 ± 0.38
AOPP-P [μmol/l]	57.63 ± 22.53^a,b^	59.46 ± 21.98^a,b^	30.85 ± 12.95	25.93 ± 10.31
Alb-S [g/l]	50.67 ± 8.58	52.52 ± 8.89	53.14 ± 6.62	51.00 ± 8.07
AOPP/albumin index [μmol/g]	1.09 ± 0.50^a,b^	1.14 **±** 0.46^a,b^	0.64 **±** 0.18^b^	0.49 **±** 0.17

^a^ *p* < 0.05 compared to smoking control group

^b^ *p* < 0.05 compared to non-smoking control group

^c^ *p* < 0.05 compared to non-smoking smelters

*AOPP-P* AOPP concentration in plasma; *Alb-S* Albumin concentration in serum; *MDA-P* MDA concentration in plasma; *8-OHdG-S* 8-hydroxy-2'-deoxyguanosine concentration in serum

In the plasma of smoking and non-smoking smelters, 2-fold higher of AOPP-P concentration in comparison to the smoking control group and 2.2-fold higher compared to the non-smoking control group was shown (Table 3). There were no statistically significant differences in Alb-S concentration between smoking and non-smoking smelters, and between smoking and non-smoking control group (Table 3).

AOPP/albumin index, which is expressed in micromole as the amount of AOPP per gram of albumin, was calculated. In the group of smoking smelters, 1.7-fold increase in the value AOPP/albumin index compared to the smoking control group and 3-fold increase of this parameter compared to the non-smoking control group was observed. 2.3-fold increase in the value of AOPP/albumin index in the non-smoking smelters compared to non-smoking control group was observed (Table 3).

### Correlations

In the serum of non-smoking smelters, the correlation was demonstrated between the concentration of Cu-S and Alb-S (*r* = −0.4646, *p* = 0.0103). The correlation of Pb-B and AOPP-P concentrations (*r* = 0.5219, *p* = 0.0073), and the AOPP/albumin index (*r* = 0.3689, *p* = 0.0369) in this group also were shown.

## Discussion

Tobacco smoke and environmental or occupational exposure to heavy metals can cause many health effects in organism of copper foundry workers. Depending on intensity and duration of the exposure, they can change the biological functions of organs, such as liver and kidney. Lead, arsenic, and cadmium belong to toxic agents, which can disturb function of cardiovascular system. Heavy metals can produce free radicals, which leads to pro-oxidant/antioxidant imbalance and oxidative stress. It leads to the disturbances of cellular metabolism resulting in the formation of permanent changes caused by the oxidation in the structure of lipids, proteins, and DNA [32]. The oxidation products are MDA, AOPP and 8-OHdG respectively. The association of the MDA, 8-OHdG, and AOPP formation and oxidative stress was confirmed in subsequent studies [2, 18, 26, 33–38].

It was demonstrated that occupational exposure causes an increase in serum Cu concentration in smelters (104.5 ± 15.1 μg %) compared to controls (99.7 ± 12.1 μg %) [39]. Also, in our study, the highest Cu-S concentration in the group of smoking smelters was shown. This indicates that Cu-S concentration was increased with the number of oxidizing factors, especially during exposure to heavy metals. It confirmed that Cu can be replaced through heavy metals in antioxidant enzymes, such as Cu/Zn superoxide dismutase (EC 1.15.1.1), which can result in the inhibition of antioxidant enzymes activity and an increase in oxidative stress [40]. On the other hand, in our study, there was no difference in Zn-S concentration in examined groups. It suggested the adaptation of healthy smelters to the environmental conditions, which was shown in others studies [41].

Bizoń et al. (2013) demonstrated that exposure to heavy metals causes an increase of Pb-B concentration [42]. Higher Pb-B concentration in the group of persons exposed to heavy metals (277.18 ± 119.14 μg/l) compared to the control group (32.11 ± 8.89 μg/l) was shown [42]. In other studies, a significant increase in Pb-B concentration in the group of smelters (201.2 ± 112.5 μg/l) compared to the people not exposed to this metal (37.9 ± 27.1 μg/l) was also shown [39]. In our study, a significant increase in Pb-B concentration in the groups of smelters compared to the control groups was observed. In the smoking smelters, the concentration of Pb was nearly sixfold higher in comparison to the non-smoking control group. It was also shown that Pb-B concentration in the group of smelters almost twofold exceeded the permissible level of this metal in the blood (<100 μg/l) [39, 43]. The Pb-B concentrations in the control groups were smaller than 100 μg/l. This confirms that the high Pb-B concentration was caused mainly by occupational exposure, and cigarette smoking was not the main source of Pb-B.

Cd-B and Cd-U concentrations increase during exposure to tobacco smoke, that was observed by other scientists. Bizoń et al. (2013) demonstrated an increase in Cd-B concentration of smokers compared to non-smokers, both in the control group and the group of smelters (0.43 ± 0.39 μg/l, 1.59 ± 0.79 μg/l and 1.69 ± 0.70 μg/l, respectively, for the non-smoking control group and smoking control group <20 cigarettes/day and >20 cigarettes/day and 0.54 ± 0.44 μg/l, 2.19 ± 1.51 μg/l and 3.05 ± 2.29 μg/l, respectively, for non-smoking and smoking smelters <20 cigarettes/day and >20 cigarettes/day) [42]. In other studies, the difference between Cd-B concentration in smokers (1.3 μg/l) and non-smokers (0.4 μg/l) was shown [44]. Madeddu et al. (2011) also demonstrated that cigarette smoking causes an increase of the Cd-B concentration (0.46 and 0.29 μg/l, respectively, for smokers and non-smokers) [45]. In this study, nearly fivefold higher of Cd-B concentration in the group of smokers compared to non-smokers (both smelters and the control group) was shown. Cd-B concentration in the group of smokers (both smelters and the control group) exceeded about threefold the value of permissible level (<0.5 μg/l) [39, 43]. In the group of non-smokers (both smelters and the control group), it did not exceed the permissible value. This emphasizes the importance of the influence of cigarette smoking on Cd-B concentration. It was observed higher Cd-U concentration in the smoking groups compared to non-smoking groups (both smelters and the control group). It was noted a higher Cd-U concentration in the group of smoking smelters compared to the smoking control group. It can suggest that occupational exposure to heavy metals accompanying exposure to tobacco smoke raised Cd-U concentration. This data confirmed that the main factor influencing on the Cd-B and Cd-U concentration is cigarette smoking. Occupational exposure to this metal seems to have lower importance on its concentration in the human body than cigarette smoking.

An increase of As-U concentration in the group of persons occupationally exposed to heavy metals (18.9 ± 22.2 μg/g creatinine) in comparison to the control group (3.5 ± 3.0 μg/g creatinine) was observed [39]. Bizoń et al. (2013) also have shown higher As-U concentration in smelters compared to the control group [42]. Our study confirmed the influence of occupational exposure on As-U, because in the groups of smelters (smokers and non-smokers) more than fourfold an increase in As-U concentration compared to the control groups was shown. It was also observed that in the group of smelters, As-U concentration exceeded the value of permissible level (<10 μg/g creatinine) [39].

The concentrations of Ca and Mg macroelements, which values have a significant impact on the reduction of the penetration capability of some toxic metals, such as Cd and Pb were also determined. The demonstrated differences in Ca-S concentration between the groups of smelters and control groups (both smokers and non-smokers) confirm that exposure to heavy metals causes a decrease Ca-S concentration. These results are similar to the study made by Kossowska et al., where was demonstrated a difference between Ca-S concentration in the group of smelters (90.4 ± 7.3 μg/ml) and the control group (99.3 ± 4.1 μg/ml) [39]. In our study, the influence of occupational exposure and cigarette smoking on Mg-S concentration was not observed. It is known that Mg has antioxidant properties, scavenging oxygen radicals, possibly by affecting the rate of spontaneous dismutation of the superoxide ion [46]. It was shown that Mg can inhibit lipoprotein oxidation and enhances the antioxidant enzymes activity, which reduces oxidative stress [47]. In our study, this effect was not observed. There was no difference in Mg-S concentration between examined groups. It can suggest that lipids peroxidation and protein oxidation in smelters were not mediated thought reduced level of Mg-S or its deficiency.

Chronic exposure to heavy metals causes their accumulation in tissues and has the influence on the human health [48–50]. It was observed an increase in the concentration of Pb-B, Cu-S, and As-U in the group of smelters, which confirms that the occupational exposure is the source of these metals. However, Cd-B concentration increases during exposure to tobacco smoke. Both occupational exposure to heavy metals and tobacco smoke intensify the oxidative stress. It results in the lipids peroxidation expressed as MDA production and/or protein oxidation and the formation of AOPP.

There was observed significantly higher plasma MDA levels (2.67 ± 0.69 μM) of workers exposed to lead than those in the control group (1.23 ± 0.61 μM) [51]. However, the results presented by Dursun et al. indicate that increased concentrations of lipid peroxidation products in the blood of persons exposed to lead is dependent not only on the concentrations of lead, but also on the age and time of exposure [51]. Other researches have also shown that MDA concentrations were elevated in the all heavy metal-treated groups compared to the control groups [52]. In this study, MDA concentrations in the group of smelters (both smokers and non-smokers) were nearly 2-fold higher compared to smoking and non-smoking control groups, which confirms that oxidative stress is generated in the result of occupational exposure in the smeltery. Increased concentration of MDA confirms the relationship between occupational exposure to heavy metals and the development of oxidative stress, and its effect on cell membranes. An increase in lipids peroxidation in smelters is a major sign of oxidative stress in the blood, which can result in the changes of tissues and organs.

Barbato et al. (2010) conducted a meta-analysis in environmental pollution exposed employees and in five studied groups, increase of 8-OHdG in the urine was observed, and in three studied group, there was no effect [53]. Garçon et al. (2007) studied the effects of chronic exposure to cadmium and lead on kidney function, they have also not observed differences in the concentration of the marker in the urine between smelters and control group (respectively, 12.7 ± 5.6 and 12.4 ± 5.9 μg/g creatinine) [54]. Wu et al. (2004) have not found any difference in the concentration of 8-OHdG in urine among smokers and people not exposed to tobacco smoke [11]. Musarrat et al. (1996) also found that adducts of 8-OHdG concentration does not correlate with smoking [55]. This study has shown no significant difference in the level of 8-OHdG concentration in none of group. This indicates that both cigarette smoking and metals exposure appear to have only a small effect on 8-OHdG level in the blood.

It should be noted that AOPP concentration depends on many physiological and environmental factors, which may influence on the examined population (age, body weight, sex, environmental pollution, cigarette smoking, and physical condition) [56–59]. The impact of additional factors, except tested factors, is so large that wrong classification to the control group or not adequate comparison of obtained results with these from other studies can make the results uncomparable. This is confirmed by the fact of the lack of standardized method for the determination of AOPP, which was noticed by other researchers [18]. In this study, examined groups were standardized in terms of sex, age, BMI, and environmental exposure to heavy metals.

There are some studies demonstrating the impact of cigarette smoking on the formation the changes in the structure of proteins. It was shown an increase in the amount of carbonyl groups in the molecules of proteins, which are the component of AOPP. A difference in the amount of carbonyl groups was shown between the group of persons, which were intensive smokers (4.00 ± 1.31 nmol/mg protein) and the persons not exposed to tobacco smoke (0.80 ± 1.94 nmol/mg protein) [14]. However, others researchers found no differences in concentration of carbonyl groups between current and former smokers (17.9 ± 2.9 nmol/ml plasma for both groups) [5]. Also was shown no difference in the concentration of carbonyl groups between persons, which were a long-term smokers (17.9 ± 2.8 and 17.9 ± 3.1 nmol/ml plasma, respectively, for smokers 30−45 years and >45 years), and the persons, which were smokers <30 years (17.7 ± 2.7 nmol/ml plasma) [5]. In some studies, it was shown that the tobacco smoking has no influence on the structure of proteins; in others, it was shown that it can induce protein oxidation measured as the increase in the amount of carbonyl groups in their molecules. This indicates that the influence of tobacco smoking on the changes in proteins structure and the formation of AOPP are not clearly defined. In our study, it was shown no influence of tobacco smoke on an increase of AOPP-P concentration. However, AOPP-P concentration was more than twofold higher in the groups of smelters than in the control groups. This indicates an influence of occupational exposure on protein oxidation. Occupational exposure to heavy metals causes a significant increase in AOPP-P concentration. Differences in this parameter resulting from the impact of tobacco smoke on protein oxidation were less visible than resulting from occupational exposure.

Because of the fact that in the composition of AOPP is mainly the products of albumin’s oxidation; the determination of this protein in the serum of smelters was an important element of this study. On the concentration of AOPP, it has an influence not only in many factors discussed above, but also the concentration of albumin in the blood of the examined persons, which were in reference range (35–55 g/l) [60]. For this reason, the parameter better expressing the changes in AOPP concentration is its conversion per gram of albumin in the blood [61–62]. The expression of AOPP concentration as AOPP/albumin index in this study allowed to make the amount of AOPP independent from the inter-person variability in the concentration of albumin. This enables to better define an exposure to oxidation factors (heavy metals and tobacco smoke). In this study, the conversion of AOPP per gram of albumin permitted to detect a statistically significant difference in the AOPP-P concentration between smoking and non-smoking control group. This difference was not detected in the case of AOPP-P concentration expressed in micromoles per liter (using the same statistical test for both parameters). It was shown that the value of AOPP/albumin index increased with the exposure to factors causing the formation of AOPP (heavy metals and also tobacco smoke).

In this study, it was demonstrated that an important factor influencing AOPP-P concentration is occupational exposure to heavy metals, especially Cu. The inverse correlation between Cu-S and Alb-S concentration may indicate the participation of Cu in the formation of AOPP and its oxidation effect on albumin. In the group of non-smoking smelters, it was demonstrated the correlation between the concentration of Pb-B and AOPP-P, and the value of AOPP/albumin index. This may indicate that high Pb-B concentration is an oxidation factor for proteins. These results suggest that Cu and Pb play an important role in protein oxidation. Cu can generate free radicals by Fenton reaction. Additionally, Pb can replace Cu in antioxidant enzymes that causes an increase in serum Cu concentration and intensifies an oxidative stress.

In this study, it was confirmed the influence of heavy metals exposure on lipids peroxidation. It indicates that occupationally exposure can have an important role in lipid degradation and cell damage. It was not observed any effect of heavy metals exposure or cigarette smoking on oxidative modification of DNA. It was shown that cigarette smoking and occupational exposure to heavy metals have an influence on protein oxidation. These oxidizing factors of which the source is the environment seem to be important for destruction of protein molecule and limit its function.

## Conclusions

In this study, it was confirmed that tobacco smoke is the main source of Cd in the blood of smelters. The occupational exposure on heavy metals has no influence on Cd-B and Cd-U concentrations. With the increasing exposure to tobacco smoke and heavy metals, Ca concentration in serum was decreased.

Exposure to heavy metals causes an increase of heavy metals concentrations in the blood, especially Pb-B, but also Cu-S and As-U of smelters. This oxidizing factor intensifies lipids peroxidation and causes an increase in MDA concentration, but they have no influence on DNA. The intensity of AOPP formation depends on the type of factor that it causes—for smelters, occupationally exposure to heavy metals is a stronger oxidation factor than tobacco smoke. The strongest oxidation effect on the protein has Cu and Pb.
